# Accuracy of Combined Osteotomy Guides and Patient-Specific Implants in Le Fort I Segmental Osteotomy Requiring Alveolar Bone Reduction

**DOI:** 10.3390/jcm15187316

**Published:** 2026-09-20

**Authors:** Xinyu Xu, Ling Wu, Zili Li, Xiaojing Liu

**Affiliations:** Department of Oral and Maxillofacial Surgery, Peking University School and Hospital of Stomatology & National Center of Stomatology & National Clinical Research Center for Oral Diseases & National Engineering Research Center of Oral Biomaterials and Digital Medical Devices, No. 22, Zhongguancun South Avenue, Haidian District, Beijing 100081, China; xuxinyu0812@hsc.pku.edu.cn (X.X.); 1764012494@pku.edu.cn (L.W.); 8864011583@pku.edu.cn (Z.L.)

**Keywords:** patient-specific implants, segmental Le Fort I osteotomy, virtual surgical planning, orthognathic surgery

## Abstract

**Background**: Segmental Le Fort I osteotomy combined with interdental alveolar bone reduction is a technically challenging procedure, and the accuracy of patient-specific implants (PSIs) in this setting has not been well established. This study aimed to assess the three-dimensional (3D) repositioning accuracy and safety of 3D-printed osteotomy guides used in conjunction with PSIs for this complex surgical procedure. **Materials and Methods**: This retrospective consecutive case series included 19 patients who underwent segmental Le Fort I osteotomy with alveolar bone reduction in the premolar region. For all patients, 3D-printed osteotomy guides and PSIs were designed using virtual surgical planning (VSP). Postoperative CBCT reconstruction models were superimposed on the VSP models to assess accuracy based on 3D deviations of skeletal and dental landmarks, angular discrepancies, and dental arch morphology. Residual intersegmental alveolar gaps and perioperative complications were also evaluated. **Results**: Signed and absolute coordinate deviations of all landmarks were within the 2 mm reference margin. Signed coordinate deviations differed significantly among the X-, Y-, and Z-axes (*p* < 0.05), with the X-axis showing the smallest mean deviation (0.064 ± 0.753 mm). The overall Euclidean spatial error was 1.477 ± 0.519 mm. Anterior maxillary segments exhibited significantly greater sagittal and vertical deviations than posterior segments (*p* < 0.05). The overall mean residual intersegmental alveolar gap was 0.681 ± 0.153 mm, with the buccal gap at the root apex significantly larger than the corresponding palatal gap (*p* < 0.05). Two minor dentin injuries occurred, neither of which resulted in clinical pulpal or periodontal complications during the available follow-up. **Conclusions**: The combined use of osteotomy guides and PSIs facilitates accurate 3D maxillary repositioning in segmental Le Fort I osteotomy involving alveolar bone reduction.

## 1. Introduction

Orthognathic surgery is a well-established approach for correcting dentofacial deformities, with the primary goals of achieving functional occlusion and improving facial aesthetics [1]. The use of custom patient-specific titanium implants (PSIs) has gained increasing acceptance as a means of enhancing the precision of maxillary repositioning and streamlining intraoperative procedures [2,3,4,5,6,7,8,9,10]. Segmental Le Fort I osteotomy is commonly performed to address transverse maxillary discrepancies and situations in which adequate orthodontic decompensation cannot be achieved [2,3,4,5,6]. Compared with conventional one-piece Le Fort I osteotomy, the segmental approach presents greater technical challenges due to the presence of multiple dentoalveolar segments, compromised vascular supply, and the potential for soft tissue interposition between adjacent segments [3,7,8,9,10,11]. Furthermore, the divided bony segments are exposed to unfavorable mechanical forces generated by occlusal loading, potentially reducing postoperative dentoalveolar stability and increasing the risk of malposition and impaired healing [3,5,11]. Collectively, these factors may limit the accuracy of three-dimensional repositioning and contribute to postoperative instability.

Limited studies have yielded conflicting results regarding the accuracy of PSIs in segmental Le Fort I osteotomy. Kuehle et al. [12] reported mean anteroposterior and vertical deviations of −0.63 ± 1.62 mm and −1.39 ± 1.59 mm, respectively, with PSI use. Rios et al. [13] also reported high positional accuracy of PSIs, with mean absolute deviations between the postoperative maxillary position and the virtual surgical plan of 0.59, 0.74, and 0.56 mm along the x-, y-, and z-axes, respectively. In contrast, a 60-patient randomized controlled trial by Diaconu et al. [14] found no significant differences in 3D accuracy between conventional and PSI-assisted surgery across any of the three axes (all *p* > 0.05).

Notably, previous studies have largely excluded patients requiring interdental alveolar bone reduction. Complete removal of bony interference while maintaining sufficient intersegmental bone stock is essential for precise segmental repositioning, restoration of the dental arch, and long-term skeletal stability. However, the accuracy of PSI-assisted surgery in segmental Le Fort I osteotomy involving alveolar bone reduction remains unclear.

Therefore, this study evaluated the three-dimensional repositioning accuracy and clinical safety of 3D-printed osteotomy guides combined with PSIs in segmental Le Fort I osteotomy requiring alveolar bone reduction. The findings may provide clinical evidence for the use of this digital workflow in complex segmental orthognathic surgery.

## 2. Materials and Methods

### 2.1. Study Design and Patient Enrollment

This single-center, retrospective consecutive case series was conducted using a prospectively maintained institutional orthognathic surgery database. Patients with dentofacial deformities who underwent orthognathic surgery at the Department of Oral and Maxillofacial Surgery, Peking University School and Hospital of Stomatology, between September 2025 and June 2026, were screened for eligibility. All eligible patients treated during the study period were consecutively included without additional sample size estimation or case matching, thereby minimizing potential selection bias associated with retrospective studies. This study was conducted in accordance with the principles of the Declaration of Helsinki (revised in 2013). The study protocol was reviewed and approved by the Institutional Review Board of Peking University School and Hospital of Stomatology (Approval No. PKUSSIRB-2026124115). Because of the retrospective study design, the ethics committee waived the requirement for additional written informed consent. All clinical and imaging data were anonymized before analysis to ensure patient confidentiality.

All surgical procedures were performed by a single senior surgeon with more than 10 years of specialized experience in orthognathic surgery, thereby minimizing inter-surgeon variability. Digital surgical planning files, intraoperative surgical records, postoperative follow-up information, and radiographic data were obtained from the hospital’s integrated electronic medical record (EMR) system and institutional digital orthognathic surgery database. During surgery, a 3D-printed guide was used to facilitate osteotomy and hole drilling, followed by internal fixation with customized PSIs.

Patients were retrospectively included according to the following criteria: (1) age 18–45 years at the time of surgery; (2) diagnosis of a dentofacial deformity requiring segmental Le Fort I osteotomy, with preoperative virtual surgical planning (VSP) identifying the need for bone removal to eliminate intersegmental bony interference; (3) use of a standardized intraoperative workflow involving a 3D-printed osteotomy guide for osteotomy execution and a PSI system for segment repositioning and rigid fixation; (4) complete surgical documentation, including virtual surgical plans and intraoperative surgical records, available in the institutional database; and (5) paired, high-quality, large-field-of-view CBCT scans obtained using the same scanner at two standardized time points: within 2 weeks before surgery for virtual surgical planning and within 1 week after surgery for postoperative accuracy assessment.

Patients were excluded if they met any of the following criteria: (1) moderate-to-severe periodontitis, generalized alveolar bone resorption, or active odontogenic infection before surgery; (2) secondary dentofacial deformities associated with maxillofacial trauma, benign or malignant jaw tumors, odontogenic cysts, cleft lip and/or palate, or congenital syndromic conditions; (3) documented intraoperative deviations from the archived preoperative VSP, including unplanned changes in the osteotomy extent, omission of planned bone removal, or modification of the original PSI fixation plan; or (4) incomplete or poor-quality archived imaging or medical records that precluded reliable three-dimensional deviation assessment.

All consecutive patients who met the inclusion and exclusion criteria during the study period were enrolled. Therefore, no formal sample size calculation was performed.

### 2.2. Treatment Process

All enrolled patients underwent a standardized, fully digital workflow for preoperative orthognathic surgical planning. Preoperative CBCT scans were acquired using a NewTom VGi evo system (Cefla S.C., Verona, Italy) with a field of view of 16 × 16 cm and an isotropic voxel size of 0.3 mm. The imaging volume extended from the glabella to the submental region. The acquired DICOM data and digital intraoral scans (TRIOS, 3Shape, Copenhagen, Denmark) were imported into a dedicated virtual surgical planning platform (IVSPlan, version 1.0.25, Weispace, Beijing, China) for data integration and three-dimensional reconstruction.

**Figure 1:**
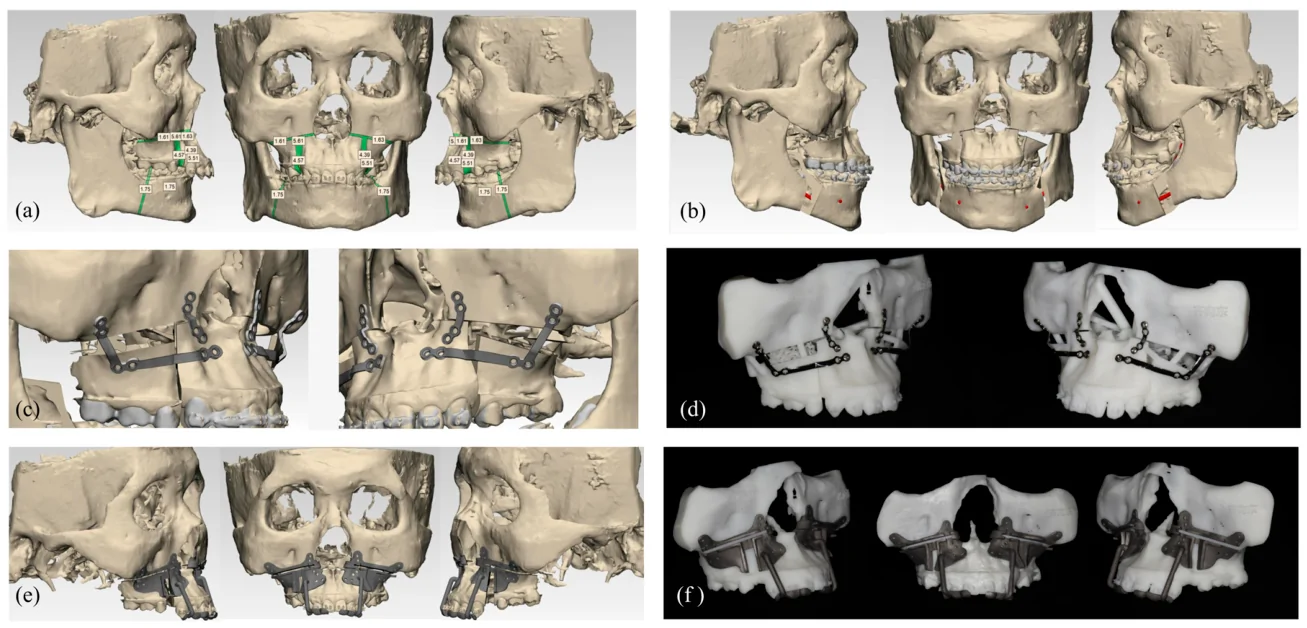
Preoperative planning, virtual surgical design, and 3D-printed osteotomy guides and PSIs in orthognathic surgery. (**a**) Preoperative skull model showing the planned osteotomy lines and bone resection sites. (**b**) Postoperative skull model. (**c**) Design of the PSIs. (**d**) Physical model of the 3D-printed PSIs. (**e**) Design of the osteotomy guides. (**f**) Physical model of the 3D-printed osteotomy guides.

The virtual surgical plan was collaboratively finalized by the attending orthodontist and the senior orthognathic surgeon responsible for performing the surgery. The VSP process comprised delineation of the Le Fort I osteotomy line, planning of maxillary segmentation, establishment of the final occlusion, removal of intersegmental bony interference, and individualized three-dimensional repositioning of each alveolar segment to achieve the intended skeletal rotation and dental arch reconstruction (Figure 1a,b). After validation of the surgical plan, a customized osteotomy guide was digitally designed to reproduce the planned osteotomy level and extent and subsequently fabricated using 3D printing for intraoperative application (Figure 1c).

#### 2.2.1. Digital Design of PSIs

Following validation of the surgical plan, the osteotomy guide was digitally designed to reproduce the planned osteotomy level and extent and then fabricated by 3D printing technology (Figure 1c,d). The guides were manufactured from TC4 (Ti-6Al-4V) using direct metal laser sintering by EOSINT M280 (EOS GmbH, Krailling, Germany). The printing parameters were set as 1200 mm/s of scan speed, 0.03 mm of layer thickness, and 190 W of laser power. After fabrication, the osteotomy guides were sterilized before surgery.

Based on the validated segmental maxillary position established through the VSP workflow, customized PSIs were separately designed for rigid internal fixation. In total, four patient-specific titanium plates were digitally contoured to conform to the native anatomical morphology of the maxillary skeleton. The two L-shaped plates were designed with elongated arms extending antero-posteriorly along the maxillary skeleton. Where the plate path intersected the projected dental root contours, the L-shaped framework was anatomically adapted to follow the external radicular profile while maintaining a uniform 0.5-mm clearance from the root surface. These interdental plates primarily stabilized the segmental alveolar units in the transverse and anteroposterior directions. The remaining two C-shaped plates were contoured to conform to the anatomical region surrounding the piriform aperture, providing additional anterior skeletal support during premaxillary rotation while avoiding compression of the adjacent nasolabial soft tissues and peripheral neurovascular structures.

All 3D-printed osteotomy guides and customized titanium PSIs were fabricated by the manufacturer (Tianqi Additive, Chengdu, China) using metal additive manufacturing in accordance with the finalized digital design files (Figure 1e,f). None of the PSI plates underwent manual bending or intraoperative modification during surgery. All patients in the present study were treated using four PSI plates.

#### 2.2.2. Surgical Procedures

**Figure 2:**
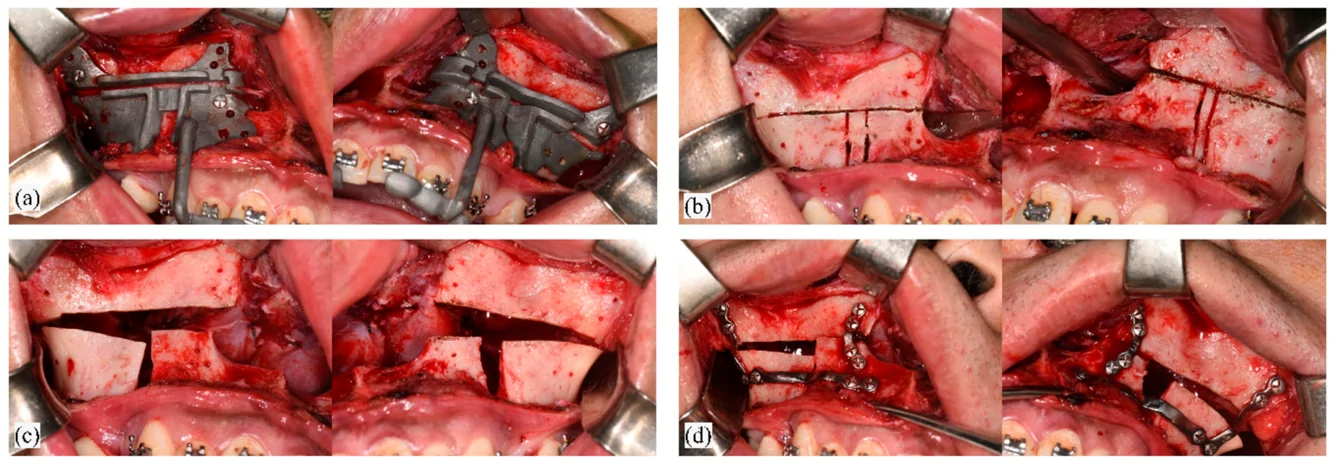
Intraoperative application of patient-specific osteotomy guides and fixation in orthognathic surgery. (**a**) Placement of the osteotomy guides. (**b**) Preparation of the osteotomy lines and screw holes. (**c**) Osteotomy and removal of bony interferences. (**d**) Placement of the PSIs and fixation of the segmental bone units.

All procedures were performed by a senior surgeon following a standardized surgical protocol. After exposure of the maxilla, the customized osteotomy guide was positioned to guide the planned segmental Le Fort I osteotomy lines and predrill the screw holes for subsequent PSI fixation (Figure 2a). The guide was then removed, and the Le Fort I osteotomy and maxillary segmentation were completed (Figure 2b). In accordance with the VSP, planned bony interferences between the dentoalveolar segments and along the palatal floor were removed (Figure 2c). The segmental bone units were subsequently repositioned and stabilized using bilateral PSIs (Figure 2d). If the predrilled bone holes did not accurately align with the corresponding screw holes of the PSI, the implant was removed to assess for soft tissue interposition along the lingual surface or palatal floor. An intermediate occlusal splint was then placed to verify whether the repositioned dental arch corresponded to the virtual surgical plan. Once the maxillary dentition was fully seated in the splint, all segmental maxillary bone units were repositioned to their planned positions. Following maxillary fixation, mandibular osteotomy and fixation were performed in accordance with the virtual surgical plan. Autogenous bone grafting was performed when the intersegmental gap exceeded 2 mm.

#### 2.2.3. Postoperative Image Acquisition

Postoperative CBCT scans were routinely obtained within 1 week after surgery as part of the standard clinical follow-up protocol, using the same CBCT system employed for preoperative imaging. The resulting images were reconstructed into three-dimensional models and subsequently used for image registration and accuracy assessment by comparison with the preoperative virtual surgical plan.

### 2.3. Outcome Measurements

To comprehensively assess the clinical performance of the proposed digital workflow, postoperative positioning accuracy was designated as the primary outcome, whereas residual intersegmental alveolar gap and postoperative complications were evaluated as secondary outcomes. Positioning accuracy was assessed based on angular measurements, three-dimensional coordinate deviations, and dental arch morphology. The residual intersegmental alveolar gap was measured on postoperative CBCT scans at the interdental osteotomy sites following definitive fixation, with measurements obtained at both the alveolar crest and apical levels. Postoperative complications were documented during the early postoperative follow-up period.

#### 2.3.1. Positioning Accuracy

##### Model Registrationa

The preoperative virtual surgical planning model was designated as T0, whereas the postoperative three-dimensional model reconstructed from CBCT scans acquired within 1 week after surgery was designated as T1. Three-dimensional analyses and model registration were performed using IVSP Image Trial software (version 1.0.24.36, IVSPlan, Beijing, China).

The T1 model was manually registered to the planned T0 model using stable, non-operated craniofacial structures, including the frontal bone and bilateral zygomatic bones. The same predefined anatomical regions were used for registration in all patients. An initial alignment was performed using three reference landmarks located at the bilateral zygomatic regions and the nasion, followed by surface-based best-fit registration using the predefined stable craniofacial region. The final registration was performed using a surface-based best-fit registration algorithm. After registration, the two models were aligned within a common three-dimensional coordinate system, allowing quantitative evaluation of positional discrepancies between the planned and postoperative maxillary segments.

##### Coordinate System

**Figure 3:**
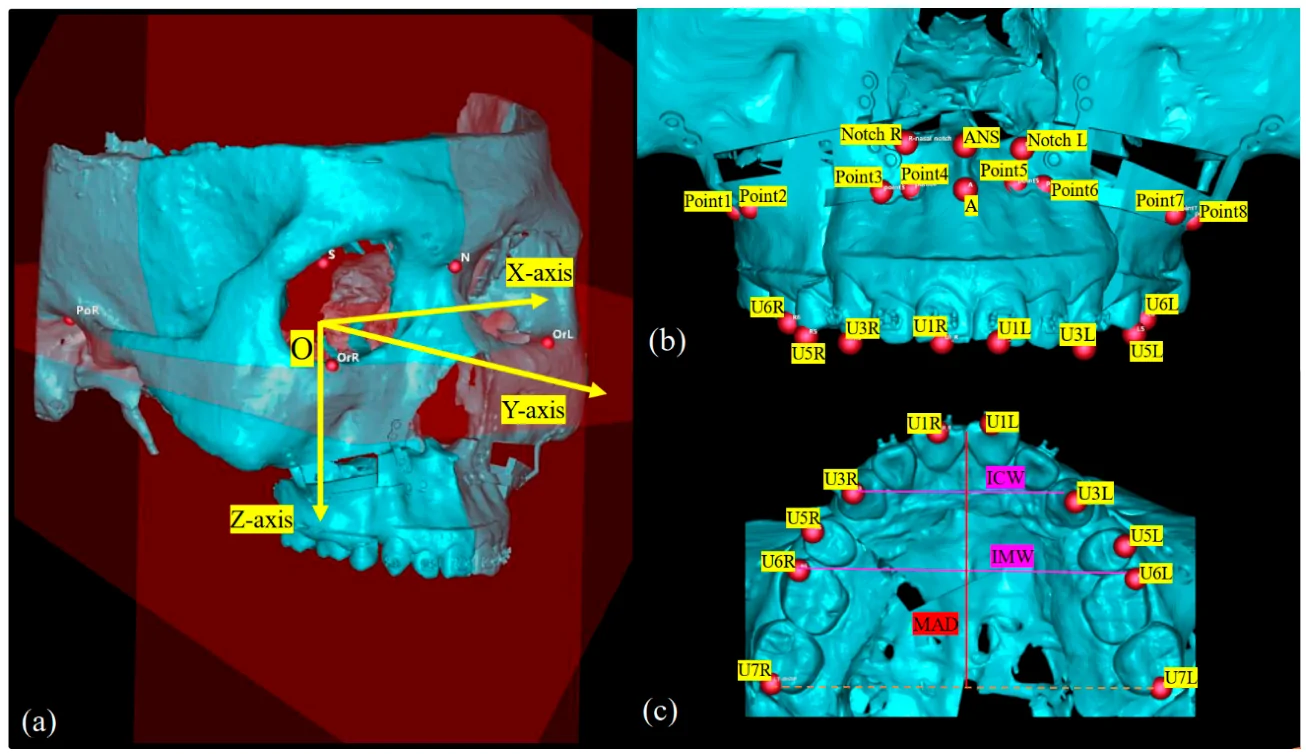
Schematic diagram of the cephalometric coordinate system and anatomical landmarks. (**a**) Coordinate system; (**b**,**c**) landmark points and measurements of ICW/IMW/MAD.

A three-dimensional coordinate system was established using predefined craniofacial anatomical landmarks, as illustrated in Figure 3a. The Frankfort horizontal plane (FHP) was defined by the bilateral orbital points and the midpoint between the bilateral porions. The midsagittal plane (MSP) was established using the Sella (S) and Nasion (N) landmarks and oriented perpendicular to the FHP. The coronal plane (CP) was established as the plane passing through the Sella point and oriented perpendicular to both the FHP and MSP. The intersection of the FHP, MSP, and CP was designated as the origin (O) of the three-dimensional coordinate system.

Accordingly, the X-axis was defined by the intersection of the FHP and CP, the Y-axis by the intersection of the FHP and MSP, and the Z-axis by the intersection of the MSP and CP. Positive values indicated leftward, anterior, and inferior displacement along the X-, Y-, and Z-axes, respectively, whereas negative values indicated rightward, posterior, and superior displacement.

##### Three-Dimensional Cephalometric Measurements

The angular parameters assessed included the SNA angle, the angle formed between the long axis of the right maxillary central incisor (U1R–U1A) and the Frankfort horizontal plane (U1–FHP), and the angle between the occlusal plane and the Frankfort horizontal plane (OPA).

Three-dimensional coordinates (X, Y, and Z) were obtained for the following anatomical landmarks: subspinale (A), anterior nasal spine (ANS), and bilateral maxillary central incisor edge points (U1L and U1R), canine cusp tips (U3L and U3R), second premolar buccal cusp tips (U5L and U5R), mesiobuccal cusp tips of the first molars (U6L and U6R), piriform aperture points (Notch L and Notch R). Eight predefined screw-hole reference points on the PSIs (Point 1–8) were also obtained to evaluate the maxilla reposition accuracy. For subgroup analyses, these landmarks were grouped according to their anatomical location. The anterior maxillary segment comprised point A, ANS, U1L/R, U3L/R, Notch L/R, and PSI screw-hole reference Points 3–6. The posterior maxillary segment included U5L/R, U6L/R, and PSI screw-hole Points 1, 2, 7, and 8. This anatomical classification was used to evaluate differences in positioning accuracy between the anterior and posterior maxillary segments. For each landmark, the signed coordinate deviation was determined by calculating the difference between the postoperative measured coordinates and corresponding preoperative planned coordinates (postoperative position − planned position) along the X-, Y-, and Z-axes. Positive and negative values represented deviations in opposite directions within the defined coordinate system. Absolute coordinate deviation was calculated as the absolute value of the signed deviation and was used to quantify the magnitude of positional error along each individual axis. Euclidean spatial error was calculated as the square root of the sum of the squared deviations along the three coordinate axes and represented the overall magnitude of three-dimensional positional error.

Dental arch morphology was assessed using three parameters: intercanine width (ICW), intermolar width (IMW), and maxillary arch depth (MAD).

Detailed definitions of all landmarks and measurement procedures are presented in Table 1, and schematic representations are shown in Figure 3b,c.

#### 2.3.2. Assessment of Residual Intersegmental Alveolar Gap

**Figure 4:**
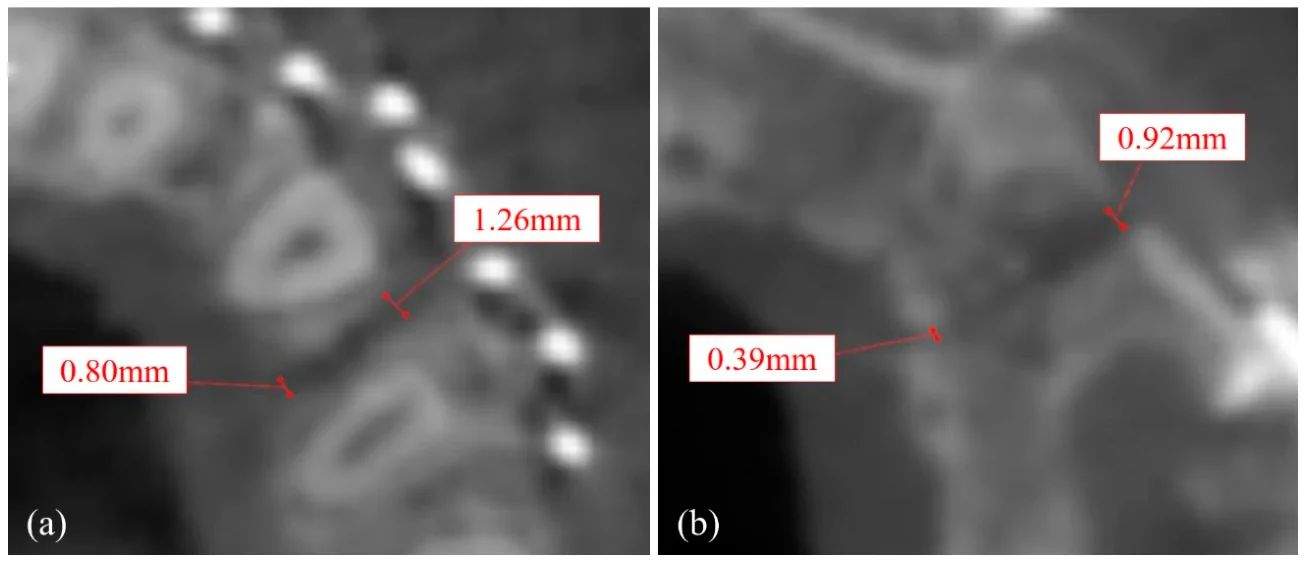
Measurement of the residual intersegmental alveolar gap. (**a**) Measurement of the buccal and palatal gaps at the alveolar crest level. (**b**) Measurement of the buccal and palatal gaps at the root apex level.

To assess the adequacy of alveolar bone reduction and the spatial relationship between adjacent dentoalveolar segments after definitive fixation, the residual intersegmental alveolar gap was evaluated using postoperative CBCT images. Measurements were obtained on coronal sections oriented perpendicular to the interdental osteotomy plane. The linear distance between the opposing osteotomy surfaces was measured at four predefined sites: the buccal and palatal aspects of the alveolar crest (crestal buccal gap and crestal palatal gap) and the buccal and palatal aspects of the root apex (apical buccal gap and apical palatal gap) (Figure 4).

#### 2.3.3. Intraoperative and Postoperative Complications

To evaluate the clinical safety of the proposed digital workflow, intraoperative and postoperative complications were retrospectively assessed through a review of electronic medical records, operative reports, postoperative CBCT images, and routine follow-up documentation. The evaluated complications included unfavorable maxillary fractures, uncontrolled intraoperative bleeding, osteotomy guide-related failures, fixation-related complications (including plate fracture, screw loosening, or PSI failure), and dental complications. Dental complications comprised tooth root injury identified on postoperative CBCT images and other clinically documented adverse dental events, such as tooth discoloration, abnormal tooth mobility unrelated to orthodontic treatment, and tooth loss. Other evaluated complications included surgical site infection, palatal fistula, wound dehiscence, maxillary segment instability, and unplanned reoperation. All complications were retrospectively assessed based on available clinical and radiographic records from routine early postoperative follow-up within 3 months after surgery.

#### 2.3.4. Measurement Reliability

Intra-observer reliability was assessed by randomly selecting 20% of the study sample for repeat measurements by the same examiner two weeks after the initial assessment. Inter-observer reliability was assessed using four patients, who were independently remeasured by a second experienced examiner blinded to the measurements of the first examiner. Relative reliability was evaluated using a single-measure intraclass correlation coefficient (ICC), based on a two-way random-effects model for absolute agreement. The repeatability assessment included both model registration and landmark identification. Additionally, to quantify the absolute random measurement error, the Dahlberg error was calculated.

### 2.4. Statistical Analysis

No missing data were identified for any of the variables included in the analysis. Consequently, no specific methods for handling missing observations (e.g., imputation or case-wise deletion) were applied.

Statistical analyses were conducted using SPSS Statistics version 27.0 (IBM Corp., Armonk, NY, USA). The Shapiro–Wilk test was used to assess the normality of continuous variables. Normally distributed continuous data are reported as mean ± standard deviation (SD), while non-normally distributed data are presented as median with interquartile range (IQR). Categorical variables are summarized as frequencies and percentages. Ninety-five percent confidence intervals (95% CIs) were calculated for the mean of all measurement variables. Parametric 95% CIs were calculated for normally distributed variables, whereas bootstrap 95% CIs were used for non-normally distributed variables.

Positional errors along the X-, Y-, and Z-axes were analyzed using linear mixed-effects models (LMMs) to account for clustering of multiple anatomical landmark measurements within individual patients.

Signed coordinate deviations were analyzed separately as dependent variables, with coordinate axis (X, Y, and Z) specified as a fixed effect and patient included as a random intercept. Estimated marginal means with corresponding 95% CIs were calculated for each coordinate axis.

Overall signed coordinate deviation was additionally estimated using the same LMM framework, with coordinate axis as a fixed effect and patient as a random intercept. The overall estimated mean and corresponding 95% CI were obtained from the model.

To compare positional errors between the anterior and posterior segments, separate LMMs were fitted for the signed coordinate deviations along the X-, Y-, and Z-axes, with segment (anterior versus posterior) specified as a fixed effect and patient included as a random intercept.

Overall Euclidean spatial error was additionally estimated using the same LMM framework, with Euclidean error as the dependent variable and patient as a random intercept. The overall estimated mean and corresponding 95% CI were obtained from the model.

All LMMs were estimated using restricted maximum likelihood (REML).

For residual intersegmental alveolar gaps, measurements from multiple osteotomy sites within each patient were averaged at each measurement location to obtain patient-level mean values. Buccal and palatal gaps were compared separately at the alveolar crest and root apex using paired-samples t-tests or Wilcoxon signed-rank tests, as appropriate based on the distribution of paired differences. Overall mean residual intersegmental gap was calculated for each patient by averaging the four measurement locations (crest-buccal, crest-palatal, apex-buccal, and apex-palatal). Patient-level mean values were used as the unit of analysis.

All statistical tests were two-sided, with *p* < 0.05 considered indicative of statistical significance.

## 3. Results

The study included 19 patients, comprising 9 males and 10 females, with a mean age of 26.95 ± 7.22 years. All patients underwent segmental Le Fort I osteotomy supported by PSIs and 3D-printed osteotomy guides, along with interdental alveolar bone removal in the premolar region. All patients underwent autogenous bone grafting at the Le Fort I osteotomy lines. In addition, interdental bone grafting was performed at 22 of the 45 interdental osteotomy sites. The demographic and surgical characteristics of the cohort are presented in detail in Table 2.

The intra-observer and inter-observer reliability analyses demonstrated excellent reproducibility. The ICC estimates were accompanied by 95% CIs ranging from 0.891 to 0.991 for intra-observer reliability and from 0.821 to 0.972 for inter-observer reliability. The Dahlberg error was 0.315 mm for three-dimensional linear measurements and 0.473° for angular measurements, indicating minimal random measurement error.

### 3.1. Three-Dimensional Coordinate Accuracy of Anatomical Landmarks

**Figure 5:**
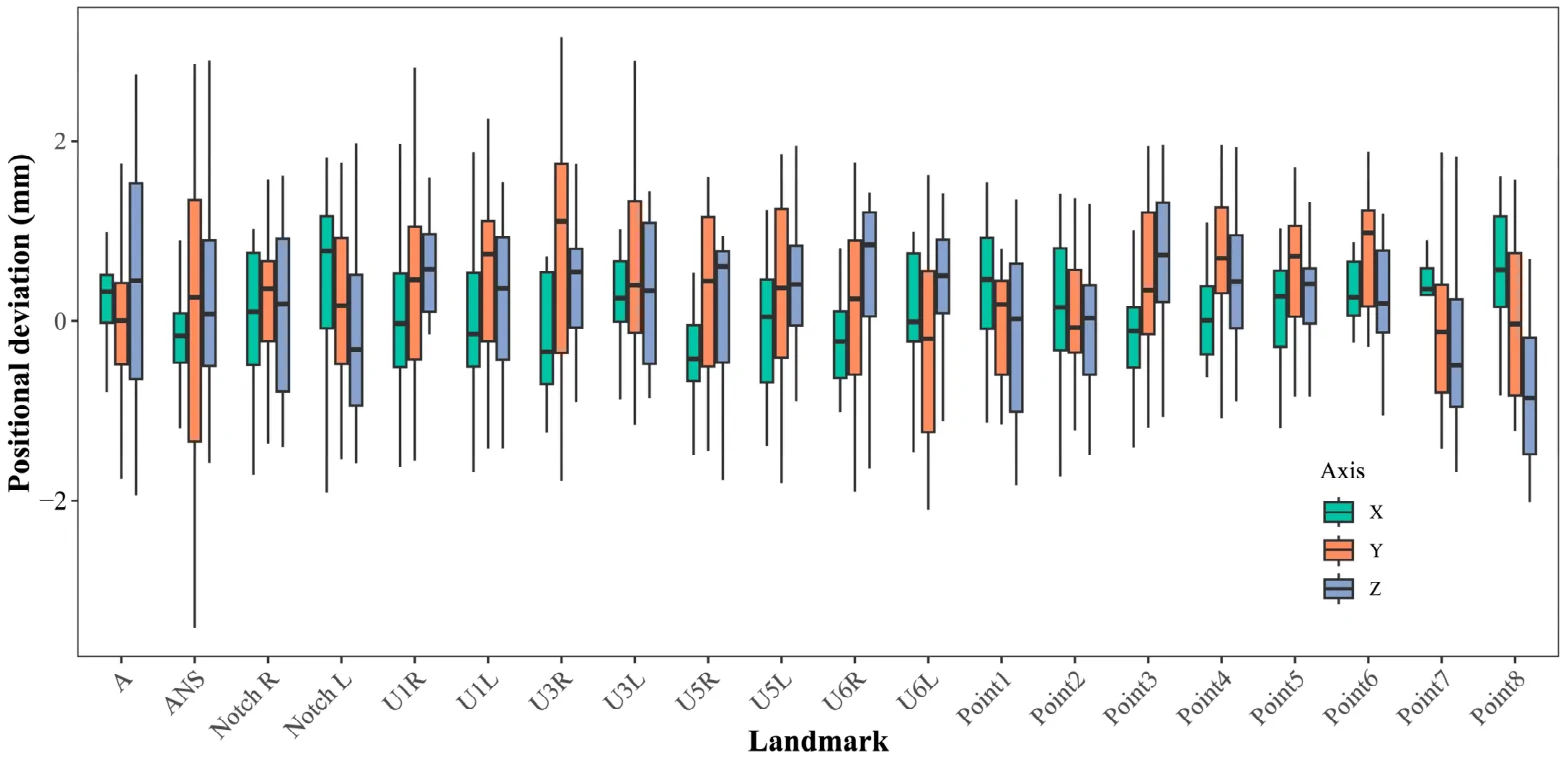
Signed coordinate deviations of all anatomical landmarks.

The signed coordinate deviations observed at all anatomical landmarks are presented in Table 3 and Figure 5. For the X-, Y-, and Z-axes, the 95% CIs of the measured deviations were all contained within the range of −2 to 2 mm.

Comparison of signed coordinate deviations along the X-, Y-, and Z-axes across all anatomical landmarks (Table 4) showed significant differences among the three axes (*p* < 0.05). Based on the estimated marginal means, the X-axis showed high precision with a mean error of 0.064 ± 0.753 mm (95% CI: −0.037 mm to 0.165 mm), which was not significantly different from zero. However, both Y and Z axes exhibited significant positive biases, with lower bounds of their 95% CIs exceeding zero (0.170 mm and 0.089 mm, respectively). Across all anatomical landmarks, overall signed coordinate deviation was 0.175 ± 0.030 mm, with a 95% CI of 0.117–0.233 mm.

To further characterize the magnitude of positional discrepancies while avoiding potential cancellation between positive and negative deviations, absolute coordinate deviations were additionally analyzed (Table 5). For the X-, Y-, and Z-axes, the 95% CIs of the absolute deviations were all within 0 to 2 mm. The Euclidean spatial errors were further assessed in Table 6; the mean or median errors were generally below 2 mm across the evaluated landmarks. The corresponding 95% CIs were entirely within the 0–2 mm range for most landmarks, although the upper limits slightly exceeded 2 mm for point A, ANS, Notch L/R and U6L. Across all anatomical landmarks, overall Euclidean spatial error was 1.477 ± 0.519 mm, with a 95% CI of 1.177–1.776 mm.

### 3.2. Anterior Versus Posterior Segment Accuracy

The three-dimensional coordinate errors of anatomical landmarks in the anterior and posterior segments are summarized in Table 7. No significant difference in X-axis positional errors was observed between the anterior and posterior segments (*p* = 0.659). However, significant differences were identified in the Y- and Z-axis errors (*p* < 0.05), with larger deviations observed in the anterior segment.

### 3.3. Angular Measurements and Dental Arch Morphology

The distributions of measurement errors for angular and dental arch morphology parameters are presented in Table 8. The 95% confidence intervals for all assessed parameters encompassed zero, with no significant systematic errors detected for any measurement.

### 3.4. Residual Intersegmental Alveolar Gap

Residual intersegmental alveolar gaps following fixation were evaluated at the alveolar crest and root apex levels using postoperative CBCT images. Residual intersegmental alveolar gaps following fixation were evaluated at the alveolar crest and root apex levels using postoperative CBCT images. As shown in Table 9, the 95% CIs of the patient-level mean gaps were entirely within 0–1 mm. At the alveolar crest level, no significant difference was observed between the buccal and palatal gaps (*p* = 0.216). At the root apex level, the buccal gap was significantly larger than the palatal gap (*p* = 0.009). The overall mean residual intersegmental gap across the four measurement locations was 0.681 ± 0.153 mm (95% CI, 0.602–0.759 mm).

### 3.5. Intraoperative and Postoperative Complications

No major intraoperative complications, including uncontrolled bleeding, palatal fistula, unfavorable maxillary fractures, or osteotomy guide-related failures, were encountered. Similarly, no cases of screw loosening, plate fracture, or PSI failure occurred during the intraoperative or postoperative period. All osteotomy guides achieved satisfactory intraoperative fit and required no substantial design modifications.

**Figure 6:**
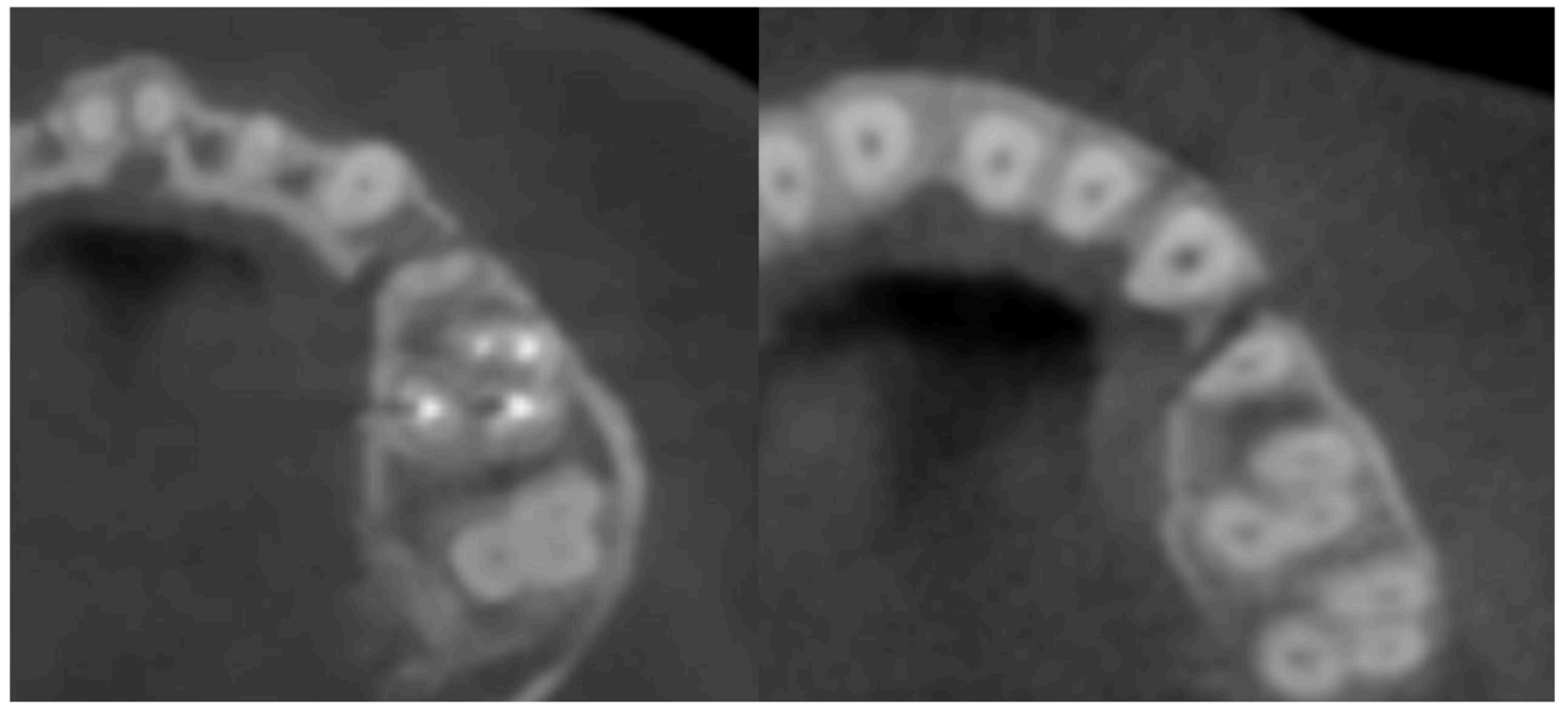
CBCT image showing minor dental injury.

Regarding dental-related complications, postoperative CBCT imaging revealed minor dentin injuries in two teeth, with an estimated defect depth of approximately 1 mm (Figure 6). Both root injuries occurred at the second premolars, with the affected areas located between the middle and apical thirds of the roots. The planned osteotomy-to-root distances at these two sites were 1.0–1.5 mm. During the period from surgery to completion of the present study, corresponding to 2.5 and 10 months of postoperative follow-up for the two affected teeth, neither tooth developed clinical signs of pulpal disease, and both responded favorably to ongoing orthodontic treatment without requiring additional dental intervention.

Postoperative wound healing was uneventful in all patients, with no cases of surgical site infection, wound dehiscence, or maxillary segment instability. No patient required unplanned reoperation.

## 4. Discussion

This study analyzed the accuracy of maxillary repositioning in PSI-assisted segmental Le Fort I osteotomy combined with alveolar bone reduction. In addition to conventional skeletal landmarks, such as point A and ANS, which have been widely used in previous studies [3,13,15,16,17], the repeatability of PSI screw holes and the piriform rim were selected as stable osseous references for evaluating surgical precision. Compared with commonly used surface-based root mean square (RMS) deviation, the landmark-based three-dimensional coordinate analysis employed in this study addressed two important methodological limitations. First, conventional anatomical landmarks may undergo morphological changes during surgery, making them difficult to reliably identify on postoperative CBCT images and potentially compromising the accuracy of postoperative assessment. In contrast, the piriform rim is relatively stable and less affected by the segmental osteotomy, while PSI screw-hole positions are specific to the digitally planned surgical construct and can be directly identified in both the preoperative virtual plan and postoperative CBCT. These characteristics make them useful complementary references for assessing surgical transfer accuracy. Second, intersegmental bone grafting performed to promote stability and remodeling, together with metallic artifacts generated by titanium PSIs, can substantially compromise surface morphology reconstruction and reduce the reliability of RMS-based assessments. In this study, bone grafting was done in all of the patients for Le Fort I osteotomy and at almost half of the interdental sites. Therefore, the evaluation approach used in this study may allow for more accurate and precise quantification of maxillary segment repositioning.

Overall, the PSI-assisted digital workflow demonstrated high accuracy in maxillary repositioning during complex segmental Le Fort I osteotomy with alveolar bone reduction. The overall Euclidean spatial error was 1.477 mm (SD, 0.519 mm), which was below the 2-mm reference threshold commonly used for positional accuracy in orthognathic surgery [13,18]. These findings indicate that the proposed protocol achieved favorable positional accuracy compared with conventional PSI-assisted segmental approaches [13]. Notably, the mean deviation of 1.63 mm (SD, 1.73 mm) reported by Diaconu et al. [14], along with the sagittal and vertical displacements described by Kuehle et al. [12], was greater than the corresponding measurements observed in the present study. The favorable accuracy observed in this cohort may theoretically be related to improved removal of intersegmental osseous interference; however, this mechanism cannot be established from the present uncontrolled study.

Nevertheless, direct comparisons across studies should be made cautiously, given differences in patient characteristics, surgical complexity, and assessment methodologies among study populations.

Further dimensional analysis demonstrated consistent patterns in the directional accuracy achieved with PSI-guided correction. In agreement with previous studies, positioning accuracy was best achieved in the transverse plane, supporting the effectiveness of PSI systems in restoring the midline during segmental Le Fort I osteotomy surgery [12,13,14]. Interestingly, the present cohort showed a slight anteroinferior displacement of the maxillary segments relative to the virtual surgical plan, contrasting with the posteroinferior drift reported in conventional segmental cases [12,14]. This difference in spatial displacement may be associated with the specific surgical requirements of alveolar bone reduction, in which surgeon-dependent palatal bone preparation may result in minor residual osseous constraints and contribute to subtle anterior segment displacement.

Subgroup analysis revealed distinct accuracy profiles between the anterior and posterior maxillary segments. Although transverse positioning accuracy was comparable between the two regions, the anterior dentoalveolar segment exhibited significantly greater sagittal [0.409 (IQR 1.511) mm vs. 0.054 (SD, 0.949) mm] and vertical [0.294 (SD, 0.976) mm vs. 0.249 (IQR, 1.487) mm] deviations. These segmental differences may be related to variations in anatomical stability and intraoperative mechanical constraints. The anterior segment has a smaller bone volume and receives less osseous and soft tissue support following segmental osteotomy. In addition, although the intraoperative occlusal splint reliably guides horizontal intercuspal alignment, it provides limited restraint against subtle anteroposterior and vertical displacement of the relatively mobile anterior segment during definitive fixation. Other factors like guide seating, PSI adaptation, soft-tissue tension, segment size, screw placement sequence, and intraoperative manipulation may also influence the repositioning accuracy. However, these potential mechanisms were not directly assessed in the present study and should therefore be considered hypotheses rather than established explanations. Nevertheless, these segment-specific positional changes remained within the predefined reference accuracy margin.

Although statistically significant differences in positional deviations were observed in some directions and between the anterior and posterior segments, the clinical significance of these relatively small deviations remains uncertain. To date, limited evidence is available regarding the relationship between the magnitude or direction of three-dimensional positional deviations and postoperative occlusion, skeletal stability, or long-term relapse following segmental Le Fort I osteotomy. Therefore, the observed deviations should be interpreted primarily as measures of surgical transfer accuracy rather than direct indicators of long-term clinical outcomes. Prospective studies with longer follow-up are warranted to determine whether these positional discrepancies have meaningful effects on occlusal function, skeletal stability, or postoperative relapse.

Assessment of the intersegmental alveolar gap objectively confirmed the adequacy of digitally guided bone reduction during complex segmental osteotomy. The resulting intersegmental gaps were consistently maintained within a narrow range, averaging 0.6–0.7 mm, thereby preventing osseous interference while minimizing unnecessary bone removal. Maintaining this balanced gap is clinically important, as a sufficiently narrow intersegmental space promotes stable osseous union and postoperative skeletal integrity, whereas inadequate bone reduction or excessive resection may impair segment adaptation and compromise long-term stability. Comprehensive quantitative evaluation at two anatomical levels (alveolar crest and root apex) and on both the buccal and palatal aspects further demonstrated a consistent distribution of intersegmental gaps, with uniformly small postoperative gaps observed across all measurement sites. Notably, the palatal apical gaps were significantly smaller than their buccal counterparts. This regional variation likely reflects differences in surgical controllability, as the buccal osteotomy is accurately guided by patient-specific osteotomy guides, whereas the palatal interdental osteotomy depends largely on the surgeon’s experience. Residual bony contact on the palatal side may have partially limited passive segment adaptation, thereby providing a plausible mechanical basis for the slight anterior sagittal displacement observed in this cohort. Residual intersegmental gaps are clinically relevant in segmental Le Fort I osteotomy because minimizing the bony defect may facilitate osseous healing and postoperative orthodontic tooth movement. Currently, there is no universally accepted international standard regarding the maximum permissible interdental osteotomy gap following segmental Le Fort I osteotomy. According to conventional fracture healing principles, fracture gaps within 1 mm are expected to heal spontaneously [19], and in some studies, gaps of 2–3 mm in maxillofacial fractures can also heal without surgical intervention [20]. Based on our clinical experience, larger gaps exceeding 2 mm tend to require bone grafting to facilitate bone healing, maintain segmental stability, and provide support for postoperative orthodontic tooth movement. However, the precise correlation between the exact gap size and subsequent recovery procedures remains unclear. Therefore, the residual gap measurements in the present study should be interpreted as an indicator of the extent of residual bony defect rather than as a definitive predictor of postoperative healing or stability.

Beyond achieving favorable repositioning accuracy, the proposed digital surgical protocol demonstrated favorable clinical outcomes, although minor dental injuries were observed. No guide-related failures, fixation complications, wound-related adverse events, or unplanned reoperations were observed during the follow-up period. Two minor intraoperative dentin injuries occurred, neither of which resulted in pulpal complications or required further dental treatment during the follow-up period. These isolated events were primarily associated with the inherent limitations of the osteotomy guides. Although the guides enable precise standardization of buccal osteotomy and screw-hole placement, they do not provide complete control over the three-dimensional trajectory of the interdental osteotomy. When the planned osteotomy is located in close proximity to the tooth root, even slight deviations from the intended trajectory may result in superficial dentin injury. This observation highlights the importance of meticulous surgical execution despite the use of digital guidance. Osteotomy guides combined with PSIs may facilitate controlled execution in complex segmental Le Fort I osteotomy, but the risk of dental or root injury cannot be completely eliminated.

Several limitations should be considered when interpreting the findings of this study. First, the absence of a control group treated with conventional titanium plates precluded direct comparison between PSI-assisted fixation and conventional fixation with respect to positioning accuracy in patients undergoing complex segmental Le Fort I osteotomy. This limitation largely stems from the absence of a standardized method for evaluating positioning accuracy in segmental Le Fort I osteotomy. Conventional surface-based assessment is confounded by intersegmental bone grafting following segmental osteotomy, whereas overall maxillary deviation measurements [12,13,14,21,22,23,24] reported in previous studies could not accurately capture the positional accuracy of both the bony segments and dentition. Second, as a single-center retrospective study, the present investigation is susceptible to selection bias and residual confounding. Furthermore, all procedures were performed by a single experienced surgeon, which enhanced procedural consistency but may limit the generalizability of the findings. Third, the relatively small sample size of 19 patients may have limited the precision and generalizability of the findings. In addition, the number of secondary statistical comparisons may have increased the risk of type I error. Therefore, the present study should be considered an exploratory and preliminary case series, and statistically significant findings from secondary analyses should be interpreted cautiously. Larger prospective studies with larger sample sizes are warranted to confirm these findings. Fourth, observer-dependent landmark identification and image registration errors inherent to three-dimensional imaging analysis could have influenced the accuracy measurements, despite standardized evaluation protocols. The observers were not blinded to the virtual surgical plan during landmark identification, which may have introduced measurement bias. However, the planned and postoperative models were not displayed simultaneously during the measurement process, which may have minimized the potential influence of direct visual comparison. Additionally, the study focused exclusively on immediate postoperative repositioning accuracy and did not evaluate long-term outcomes, including skeletal stability, bone healing, or occlusal maintenance. Thus, the short follow-up duration limits conclusions regarding the durability of the observed accuracy. Future multicenter prospective studies with larger cohorts and extended follow-up are warranted to further establish the long-term effectiveness and stability of this digitally guided surgical approach.

## 5. Conclusions

The combined use of osteotomy guides and PSIs facilitates demonstrated favorable three-dimensional agreement between postoperative maxillary position and the virtual surgical plan in segmental Le Fort I osteotomy with alveolar bone reduction. Although the anterior dentoalveolar segment exhibited slightly greater sagittal and vertical deviations than the posterior segment, the observed positional errors were generally within the 2-mm reference range commonly used in orthognathic surgery. These findings support the feasibility and favorable positional accuracy of this digitally guided workflow for complex segmental Le Fort I osteotomy; however, further comparative studies are needed to determine whether it offers advantages over conventional fixation or other digital surgical approaches.

## Figures and Tables

**Table 1:** Definition of landmarks, reference planes and measurements.

	Abbreviation	Definition
Landmark		
Sella	S	Geometric center of the sella turcica.
Nasion	N	Most anterior point of the frontonasal suture in the midsagittal plane.
Orbitale	OrL/OrR	Lowest point on the left or right infraorbital rim.
Porion	PoL/PoR	Highest point on the left or right external acoustic meatus.
Subspinale	A	Deepest point on the anterior maxilla concavity in the midsagittal plane.
Anterior nasal spine	ANS	Most anterior point of the anterior nasal spine, located at the inferior border of the piriform aperture and formed by the midline fusion of the maxillae.
Upper incisor tip	U1L/R	Most mesial point on the incisal edge of the left or right maxillary central incisor crown.
Upper incisor apex	U1A	Root apex of the right maxillary central incisor.
Upper canine cusp	U3L/U3R	Cusp tip of the left or right maxillary canine.
Upper second premolar buccal cusp	U5L/U5R	Buccal cusp tip of the left or right maxillary second premolar.
Upper first molar	U6L/U6R	Mesiobuccal cusp tip of the left or right maxillary first molar.
Upper second molar	U7L/U7R	Midpoint of the distal marginal ridge of the left or right maxillary second molar.
Apertura piriformis point	Notch L/R	Most lateral point on the margin of the left or right piriform aperture.
Implant screw-hole reference point	Point 1–8	Predetermined screw-hole locations on the PSIs attached to the tooth-bearing segment, sequentially numbered from right to left as Points 1–8.
Plane		
Frankfort Horizontal Plane	FHP	Plane defined by the bilateral OrL, OrR, and the midpoint between the bilateral porions.
Midsagittal Plane	MSP	Plane passing through S and N, perpendicular to the FHP.
Coronal Plane	CP	Plane passing through S and perpendicular to both the FHP and MSP.
Occlusal plane	OP	Plane defined by U6L, U6R and U1.
Measurement		
Maxillary intercanine width	ICW	Linear distance between the cusp tips of the left and right maxillary canines.
Maxillary intermolar width	IMW	Linear distance between the central fossae of the left and right maxillary first molars.
Maxillary arch depth	MAD	Perpendicular distance from the contact point of bilateral maxillary central incisors to the U7L-U7R line.
SNA angle	SNA	Angle formed by points S, N, and A.
Upper incisor inclination	U1–FHP	Angle between the long axis of the right maxillary central incisor (U1R–U1A) and the FHP.
Occlusal plane angle	OPA	The angle between OP and FHP.

**Table 2:** Demographic and surgical characteristics of the cohort.

Variable	Overall Cohort
Demographic and skeletal characteristics	
Age, years	26.95 ± 7.22
Sex, male:female	9:10
Sagittal skeletal pattern, patients, *n* (%)	
Class I	4 (21.1)
Class II	7 (36.8)
Class III	8 (42.1)
Maxillary surgical characteristics	
Number of maxillary segments per patient, mean ± SD	2.47 ± 0.68 (range, 2 to 4)
Type of segmental Le Fort I osteotomy, patients, *n* (%)	
2-piece	11 (57.9)
3-piece	6(31.6)
4-piece	2 (10.5)
Number of interdental osteotomy sites, total No. of sites	45
Location of interdental osteotomy sites, *n* (% of sites)	
Between central incisors	7 (15.6)
Between canine and premolar	38 (84.4)
Planned maxillary movements	
Anteroposterior movement, mm, mean ± SD	−1.27± 2.38 (range, −6.2 to 3)
Advancement, patients, *n* (%)	3 (15.8)
Setback, patients, *n* (%)	11 (57.9)
No change, patients, *n*(%)	5 (26.3)
Alveolar bone management	
Planned alveolar bone removal, mm, mean ± SD	3.58 ± 1.71 (range, 1.10 to 6.86)
Bone grafting sites	
Interdental bone grafting, total No. of sites	23
Between central incisors, sites, *n* (% of sites)	3 (13.0)
Between canine and premolar, sites, *n* (% of sites)	20 (87.0)
Le Fort I osteotomy line grafted, *n* (% of sites)	38 (100)
Associated procedures	
Associated mandibular osteotomy, patients, *n* (%)	19(100)
Bilateral sagittal split osteotomy, patients, *n* (%)	18 (94.7)
Subapical osteotomy, patients, *n* (%)	2(10.5)
Genioplasty, patients, *n* (%)	12 (63.2)
Immediate post-operation follow-up, days, mean ± SD	3.67 ± 1.37 (range, 2 to 7)

**Table 3:** Signed coordinate deviations of all anatomical landmarks.

	X/mm			Y/mm			Z/mm		
	**Mean ± SD**	**Median (IQR)**	**95%CI**	**Mean ± SD**	**Median (IQR)**	**95%CI**	**Mean ± SD**	**Median (IQR)**	**95%CI**
A	0.161 ± 0.697	-	(−0.231, 0.535)	−0.085 ± 1.069	-	(−0.715, 0.433)	0.484 ± 1.298	-	(−0.228, 1.201)
ANS	−0.032 ± 0.843	-	(−0.371, 0.475)	0.041 ± 1.644	-	(−0.903, 0.895)	0.322 ± 1.329	-	(−0.349, 1.095)
Notch R	−0.012 ± 0.885	-	(−0.476, 0.494)	-	0.361 (1.281)	(−0.482, 0.910)	0.116 ± 0.977	-	(−0.383, 0.682)
Notch L	0.448 ± 0.987	-	(−0.128, 0.944)	0.194 ± 1.021	-	(−0.356, 0.767)	−0.215 ± 0.958	-	(−0.75, 0.304)
U1R	−0.041 ± 0.895	-	(−0.559, 0.416)	0.478 ± 1.12	-	(−0.188, 0.852)	-	0.573 (1.096)	(−0.026, 0.971)
U1L	−0.038 ± 0.850	-	(−0.535, 0.386)	0.534 ± 1.141	-	(−0.151, 1.021)	0.145 ± 0.934	-	(−0.222, 0.706)
U3R	−0.226 ± 0.683	-	(−0.524, 0.184)	0.760 ± 1.334	-	(−0.026, 1.410)	0.304 ± 0.834	-	(−0.114, 0.791)
U3L	0.264 ± 0.711	-	(−0.109, 0.670)	0.455 ± 1.306	-	(−0.304, 1.116)	-	0.338 (1.788)	(−0.229, 0.709)
U5R	−0.404 ± 0.497	-	(−0.68, −0.133)	0.275 ± 1.003	-	(−0.234, 0.857)	-	0.603 (1.393)	(−0.295, 0.596)
U5L	−0.078 ± 0.724	-	(−0.51, 0.252)	0.288 ± 1.169	-	(−0.36, 0.926)	0.524 ± 0.888	-	(0.078, 1.042)
U6R	−0.195 ± 0.488	-	(−0.422, 0.096)	0.092 ± 1.075	-	(−0.449, 0.718)	-	0.845 (1.394)	(0.027, 0.998)
U6L	−0.139 ± 0.561	-	(−0.431, 0.183)	−0.172 ± 1.168	-	(−0.806, 0.478)	0.288 ± 0.870	-	(−0.204, 0.752)
Point1	0.414 ± 0.797	-	(0.109, 0.893)	−0.003 ± 0.647	-	(−0.312, 0.38)	−0.109 ± 0.952	-	(−0.657, 0.382)
Point2	0.170 ± 0.789	-	(−0.188, 0.642)	0.090 ± 0.705	-	(−0.270, 0.498)	−0.155 ± 0.775	-	(−0.594, 0.256)
Point3	−0.187 ± 0.635	-	(−0.535, 0.163)	0.441 ± 0.942	-	(−0.123, 0.868)	0.674 ± 0.832	-	(0.185, 1.083)
Point4	0.037 ± 0.708	-	(−0.320, 0.449)	0.790 ± 0.979	-	(0.219, 1.068)	0.466 ± 0.781	-	(0.062, 0.916)
Point5	0.128 ± 0.626	-	(−0.224, 0.464)	0.517 ± 0.774	-	(0.056, 0.876)	0.258 ± 0.853	-	(−0.229, 0.705)
Point6	0.199 ± 0.717	-	(−0.212, 0.573)	0.744 ± 0.698	-	(0.325, 1.021)	0.339 ± 0.915	-	(−0.204, 0.775)
Point7	0.297 ± 0.663	-	(−0.072, 0.658)	−0.096 ± 0.927	-	(−0.612, 0.408)	−0.232 ± 1.032	-	(−0.773, 0.358)
Point8	0.517 ± 0.721	-	(0.146, 0.933)	−0.043 ± 0.856	-	(−0.522, 0.42)	−0.734 ± 0.863	-	(−1.201,−0.251)

**Table 4:** Comparison of three-dimensional signed coordinate deviations across anatomical landmarks.

	Mean ± SD/mm	Median (IQR)/mm	95%CI/mm ^e^	LMM	
				*F*	*p*-Value
X	0.064 ± 0.753	-	(−0.037, 0.165)	4.131	0.016 *
Y	-	0.347(1.500)	(0.170, 0.372)		
Z	-	0.327(1.429)	(0.089, 0.291)		
overall	0.175 ± 0.030	0.256(1.271)	(0.117, 0.233)	-	-

* *p* < 0.05; ^e^ values were adjusted for patient-level random effects using a linear mixed model.

**Table 5:** Absolute coordinate deviations of all anatomical landmarks.

	X/mm			Y/mm			Z/mm		
	Mean ± SD	Median (IQR)	95%CI	Mean ± SD	Median (IQR)	95%CI	Mean ± SD	Median (IQR)	95%CI
A	0.593 ± 0.373	-	(0.400, 0.832)	-	0.484 (1.259)	(0.419, 1.225)	1.139 ± 0.745	-	(0.719, 1.544)
ANS	0.666 ± 0.49	-	(0.345, 0.882)	1.332 ± 0.906	-	(0.869, 1.92)	0.949 ± 0.958	-	(0.491, 1.583)
Notch R	0.982 ± 0.854	-	(0.492, 1.047)	-	0.754 (0.752)	(0.519, 1.5)	0.861 ± 0.426	-	(0.653, 1.137)
Notch L	0.856 ± 0.553	-	(0.587, 1.252)	0.893 ± 0.583	-	(0.581, 1.14)	0.806 ± 0.526	-	(0.54, 1.113)
U1R	0.697 ± 0.535	-	(0.412, 1.038)	0.971 ± 0.704	-	(0.522, 1.064)	0.907 ± 0.531	-	(0.552, 1.154)
U1L	0.652 ± 0.521	-	(0.326, 0.857)	0.98 ± 0.591	-	(0.614, 1.194)	0.767 ± 0.520	-	(0.411, 0.974)
U3R	0.632 ± 0.312	-	(0.434, 0.772)	1.159 ± 0.674	-	(0.742, 1.485)	0.693 ± 0.533	-	(0.385, 0.930)
U3L	0.583 ± 0.467	-	(0.343, 0.862)	0.915 ± 0.775	-	(0.452, 1.335)	0.774 ± 0.427	-	(0.52, 1.026)
U5R	0.533 ± 0.345	-	(0.355, 0.756)	0.914 ± 0.446	-	(0.751, 1.22)	-	0.754 (0.281)	(0.502, 0.949)
U5L	0.573 ± 0.427	-	(0.303, 0.805)	1.006 ± 0.614	-	(0.664, 1.32)	-	0.514 (0.721)	(0.444, 1.194)
U6R	0.428 ± 0.290	-	(0.232, 0.509)	0.864 ± 0.61	-	(0.528, 1.245)	0.923 ± 0.401	-	(0.732, 1.19)
U6L	0.460 ± 0.333	-	(0.266, 0.66)	1.019 ± 0.54	-	(0.761, 1.363)	0.793 ± 0.420	-	(0.552, 1.033)
Point 1	0.737 ± 0.491	-	(0.426, 1.002)	0.558 ± 0.296	-	(0.371, 0.719)	0.762 ± 0.549	-	(0.439, 1.023)
Point 2	0.628 ± 0.484	-	(0.383, 0.931)	0.574 ± 0.395	-	(0.381, 0.834)	0.619 ± 0.467	-	(0.367, 0.828)
Point 3	0.494 ± 0.426	-	(0.245, 0.728)	0.821 ± 0.615	-	(0.408, 1.093)	0.883 ± 0.588	-	(0.502, 1.183)
Point 4	0.526 ± 0.457	-	(0.312, 0.826)	0.952 ± 0.812	-	(0.517, 1.189)	0.733 ± 0.520	-	(0.482, 1.066)
Point 5	0.539 ± 0.317	-	(0.41, 0.755)	0.78 ± 0.486	-	(0.446, 0.948)	0.697 ± 0.531	-	(0.382, 1.008)
Point 6	0.588 ± 0.435	-	(0.321, 0.829)	-	0.976 (0.983)	(0.443, 1.033)	-	0.699 (0.877)	(0.247, 1.02)
Point 7	-	0.401 (0.463)	(0.416, 0.857)	0.753 ± 0.516	-	(0.523, 1.089)	0.857 ± 0.585	-	(0.499, 1.161)
Point 8	0.765 ± 0.427	-	(0.594, 1.054)	0.727 ± 0.416	-	(0.593, 1.013)	0.919 ± 0.482	-	(0.647, 1.199)

**Table 6:** Euclidean spatial errors of all anatomical landmarks.

	Mean ± SD/mm	Median (IQR)/mm	95%CI
A	1.703 ± 0.718	1.901 (1.154)	(1.276, 2.156)
ANS	1.812 ± 0.631	-	(1.546, 2.285)
Notch R	1.531 ± 0.718	-	(1.157, 2.017)
Notch L	1.629 ± 0.646	-	(1.221, 2.015)
U1R	1.566 ± 0.639	-	(1.117, 1.861)
U1L	1.403 ± 0.645	-	(1.003, 1.773)
U3R	1.625 ± 0.519	-	(1.323, 1.920)
U3L	-	1.428 (0.738)	(1.206, 1.874)
U5R	1.386 ± 0.400	-	(1.235, 1.671)
U5L	-	1.451 (0.800)	(1.238, 1.942)
U6R	1.455 ± 0.509	-	(1.131, 1.736)
U6L	1.800 ± 0.735	-	(1.409, 2.327)
Point 1	1.310 ± 0.576	-	(0.959, 1.650)
Point 2	1.210 ± 0.478	-	(0.958, 1.532)
Point 3	1.439 ± 0.713	-	(1.023, 1.854)
Point 4	1.461 ± 0.470	-	(1.257, 1.790)
Point 5	1.314 ± 0.506	-	(0.938, 1.551)
Point 6	-	1.262 (0.942)	(0.911, 1.630)
Point 7	-	1.315 (0.433)	(1.139, 1.826)
Point 8	1.584 ± 0.435	-	(1.364, 1.845)

**Table 7:** Comparison of three-dimensional coordinate errors between landmarks in the anterior and posterior segments.

		Mean ± SD /mm	Median(IQR) /mm	95%CI/mm ^e^	LMM	
					*F*	*p*-Value
X	anterior	0.049 ± 0.760	-	(−0.054, 0.153)	0.201	0.659
	posterior	0.086 ± 0.745	-	(−0.041, 0.213)		
Y	anterior	-	0.409 (1.511)	(0.269, 0.563)	11.815	0.002 **
	posterior	0.054 ± 0.949	-	(−0.127, 0.234)		
Z	anterior	0.294 ± 0.976	-	(0.161, 0.427)	9.060	0.016 *
	posterior	-	0.249(1.487)	(−0.129, 0.197)		

* *p* < 0.05, ** *p* < 0.01; ^e^ values were adjusted for patient-level random effects using a linear mixed model.

**Table 8:** Error analysis of angular and dental arch morphology measurements.

	Mean ± SD	Median (IQR)	95%CI
SNA/°	−0.076 ± 0.996	-	(−0.665, 0.400)
U1-FHP/°	0.266 ± 1.353	-	(−0.433, 1.045)
OPA/°	0.124 ± 1.254	-	(−0.544, 0.792)
ICW/mm	0.064 ± 0.83	-	(−0.288, 0.562)
IMW/mm	−0.314 ± 0.729	-	(−0.652, 0.120)
MAD/mm	−0.071 ± 0.864	-	(−0.532, 0.389)

**Table 9:** Error analysis of residual intersegmental alveolar gaps.

Gap		Mean ± SD/mm	Median (IQR)/mm	95%CI	*p*-Value
alveolar crest	buccal	0.711 ± 0.193		(0.612, 0.811)	0.216
	palatal	-	0.550 (0.243)	(0.488, 0.776)	
root apex	buccal	0.772 ± 0.207		(0.665, 0.879)	0.009 **
	palatal	0.607 ± 0.205		(0.502, 0.713)	
overall		0.681 ± 0.153	-	(0.602, 0.759)	-

** *p* < 0.01.

## Data Availability

To protect patient privacy, the datasets generated and/or analyzed during the current study are available from the corresponding author upon reasonable request.
